# Shrimp Extract Exacerbates Allergic Immune Responses in Mice: Implications on Clinical Diagnosis of Shellfish Allergy

**DOI:** 10.1007/s12016-024-08994-4

**Published:** 2024-05-22

**Authors:** Wai Sze Tong, Shanshan Li, Nicki Y. H. Leung, Wing Tak Wong, Ting Fan Leung, Patrick S. C. Leung, Ka Hou Chu, Christine Y. Y. Wai

**Affiliations:** 1 School of Life Sciences, The Chinese University of Hong Kong, HKSAR, China; 2 Department of Paediatrics, Faculty of Medicine, The Chinese University of Hong Kong, HKSAR, China; 3 Division of Rheumatology, Allergy and Clinical Immunology, University of California, Davis, CA USA; 4 Southern Marine Science and Engineering Guangdong Laboratory (Guangzhou), Guangzhou, China; 5 Present Address: Hong Kong Hub of Paediatric Excellence (HK HOPE), Faculty of Medicine, The Chinese University of Hong Kong, HKSAR, China

**Keywords:** Shrimp allergy, Food allergy, Mouse model, Tropomyosin, Skin prick test

## Abstract

Tropomyosin has been identified as the major cross-reactive shellfish allergen, but recent studies showed the presence of other clinically relevant allergens. This study aims at determining the allergic immune responses of mice sensitized with raw and boiled shrimp extracts in comparison to recombinant tropomyosin (rTM). Female Balb/c mice were intragastrically sensitized and challenged with raw, boiled shrimp or rTM. Systemic, cellular and humoral allergic responses were compared, while allergenicity of the extracts was also compared by skin prick test (SPT) and immunoblot on shrimp allergic subjects. We showed that rTM and shrimp extracts induced IgE- and Th2-mediated allergic responses in mice, distinguished by remarkable intestinal inflammation in small intestine across all regimens. Notably, boiled shrimp extract exhibited the highest sensitization rate (73.7% of mice developed positive TM-specific IgE response) when compared with raw extract (47.8%) and rTM (34.8%). Mice sensitized with boiled extract manifested the highest allergen-specific IgE and Th2 cytokine responses than the others. Immunoblot results indicated that tropomyosin remained the major allergen in extract-based sensitization and had stronger allergenicity in a heat-treated form comparing to untreated TM, which was in line with the SPT results that boiled extract induced larger wheal size in patients. Hemocyanin and glycogen phosphorylase were also identified as minor allergens associated with manifestation of shrimp allergy. This study shows that boiled extract enhanced sensitization and Th2 responses in agreement with the higher allergenicity of heat-treated TM. This study thus presents three shrimp allergy murine models suitable for mechanistic and intervention studies, and in vivo evidence implies higher effectiveness of boiled extract for the clinical diagnosis of shellfish allergy.

**Supplementary Information:**

The online version contains supplementary material available at 10.1007/s12016-024-08994-4.

## Introduction

Shellfish allergy affects up to 3% of the general population and is the most common trigger of food allergy among adults [1]. Prevalence of shellfish allergy is often higher in the Asia–Pacific region and coastal areas where shellfish consumption is high [2, 3]. Shellfish allergy is often lifelong, and is the leading cause of food-induced anaphylaxis [4]. While we continue to see rising rates of food allergy, including to shellfish, mouse models for identifying the mechanisms of sensitization and new immunotherapeutic interventions are important for translational medicine applications. Our group has established a murine model of shrimp allergy based on intragastric sensitization and challenge with recombinant shrimp tropomyosin (rTM) from *Metapenaeus ensis *[5], which is the major cross-reactive allergen across shellfish, as well as arthropods such as house dust mites and cockroaches. This model recapitulated the immunological changes in humans, and featured with a remarkable increase in the number of inflammatory cells within the small intestine even without repetitive intragastric challenges [6].

Although our model serves as a very useful tool for testing new therapeutic interventions [7–9], it does not fully mimic “real-world sensitization” since it focuses only on a single major allergen. Shrimp are often consumed cooked, while significant increase in IgE binding capacity was detected in heated shrimp TM (via boiling, baking, steaming and frying) comparing to raw TM [10]. Besides, our group has recently comprehended the allergenic repertoire of the black tiger prawn (*Penaeus monodon*) from oral food challenge-proven shrimp allergic subjects [11]. Nine shrimp allergens are now officially registered with the World Health Organization and International Union of Immunological Societies (WHO/IUIS). Of note, troponin C (Pen m 6) and fatty acid-binding protein (Pen m 13) are clinically important major allergens beyond TM, while sensitization to the newly identified high-molecular-weight allergen, glycogen phosphorylase (Pen m 14) is associated with positive oral food challenge. It was also demonstrated that only peanut extract but not any of its individual allergens induced expression and activity of RALDH2 in human antigen presenting cells [12]. Only peanut extract led to the production of retinoic acid to act on Th cells for inducing IL-5 and gut-homing integrin. Peanut proteins thus act as Th2-promoting adjuvant that possibly explain the potent allergenicity of peanut. Mouse model based on extract-induced allergy can therefore more comprehensively address the clinical manifestation of this disease.

In this study, we generated mouse models of shrimp allergy based on oral sensitization and challenge with raw and boiled shrimp extracts and compared their hypersensitivity responses with mice administered with rTM. These models would be of particular importance to understand the profile and allergenicity of shrimp allergens, the mechanism of shellfish allergy that could elicit severe and life-threatening allergic reactions, as well as identifying safe and effective intervention for this disease.

## Methods

### Animals

Three to four weeks old female BALB/c mice were acquired from the Laboratory Animal Services Centre of The Chinese University of Hong Kong. All mice were maintained in fully accredited facilities in the Animal Unit at the university and fed with shrimp-free diet. Ethical approval for animal experimentation was obtained from the Animal Experimentation Ethics Committee of The Chinese University of Hong Kong (Ref. No. 21–243-NIH). All animal experiments were conducted under licenses granted by the Department of Health, HKSAR Government, China.

### Preparation of Shrimp Proteins

Recombinant shrimp TM (rTM) expressed in pET30a (carrying N-terminal His-Tag/thrombin/S-Tag and C-terminal His-Tag) was prepared as previously described [13]. *Metapenaeus ensis* (greasyback shrimp) acquired from local market was used to prepare raw and boiled extracts. To prepare raw extract, the abdomen muscle isolated from five shrimp were blended in (1:1 wt/vol) phosphate-buffered saline (PBS) and sonicated for 5 min using an ultrasonic probe followed by centrifugation for 10 min at 12,000 rpm at 4 °C. Supernatant was obtained as raw shrimp extract. The boiled extracts were prepared by boiling abdomen muscle of five shrimp for 10 min in boiling water (100 oC). The samples were then sonicated and centrifuged as described above to collect the supernatant as boiled shrimp extract. Protein concentrations of raw and boiled extract, as well as rTM were determined by spectrophotometry on NanoDrop OneC (Thermo Fisher Scientific) at A280.

### Sensitization and Challenge of Mice

**Fig. 1:**
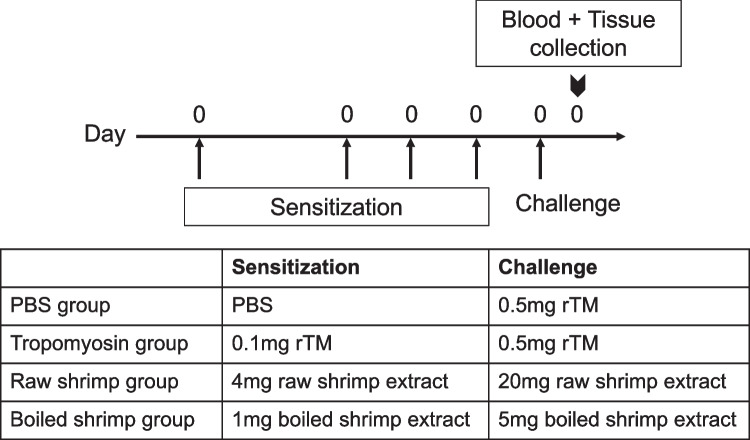
Experimental design of shrimp allergy mouse model. 3–4 weeks old BALB/c mice (n = 16–23 in each group) were sensitized intragastrically with recombinant tropomyosin (rTM), raw shrimp extract and boiled shrimp extract respectively using cholera toxin as adjuvant on days 0, 12, 19 and 26, followed by a 5-fold challenge on day 33. Mice fed with PBS served as negative control. Mice were sacrificed on day 34 that blood sample, small intestine and spleen were harvested for analysis

BALB/c mice were randomly divided into four groups: negative control, rTM group, raw shrimp group and boiled shrimp group. The experiment protocol for sensitization and challenge of the animals is shown in Fig. 1 as previously described [5]. Mice were intragastrically sensitized with 0.1 mg rTM, 4 mg (total protein content) of raw shrimp extract or 1 mg of boiled shrimp extract, respectively, on days 0, 12, 19 and 26 with 10 µg cholera toxin (CT) per sensitization. The sensitization doses were determined based on the same quantity of tropomyosin in the sensitizing agent as estimated by their relative quantity on the resolved protein gel using ImageLab (Bio-Rad). On day 33, mice sensitized with rTM, raw shrimp extract and boiled shrimp extract were challenged intragastrically with 0.5 mg rTM, 20 mg raw shrimp extract or 5 mg boiled shrimp extract, respectively. Mice in the negative control group were given PBS throughout the experiment. All mice were sacrificed on day 34 post-challenge for blood collection and harvesting of spleen and intestine. The experiment was repeated for five times, with a total of 19–23 animals per group.

### Assessment of Systemic Anaphylaxis and Diarrhea

Systemic allergic responses and condition of feces (i.e. sign of diarrhea) were evaluated 30 to 45 min after oral challenge, according to a scoring system for determining IgE-mediated responses as previously described [5, 14].

### Levels of Serum IgE Antibodies

Allergen-specific IgE levels in blood were measured by ELISA. Briefly, 96-well plates (Nunc, Thermo Scientific) were coated with 100 µl rTM, raw or boiled shrimp extract (5 µg/ml) in 0.05 M carbonate buffer overnight at 4 °C. Diluted serum samples (1:10) were incubated overnight at 4 °C after blocking the plates in 5% FBS/PBS. Bound IgE antibodies were detected using Biotin Anti-Mouse IgE (1:1000 dilution, BD Pharmingen), followed by HRP avidin D (1:1000 dilution, Vector Labs). Upon signal development with TMB substrate (BD Biosciences) and reaction termination with 0.1 M sulfuric acid, the optical density (OD) at 450 nm was measured using a microplate reader (BioTek). All assays were performed in duplicate. Positive reaction was defined as OD > 0.34 (maximum value + SD of IgE_rTM_ in the negative control group).

### Immunoblotting

Quantity of tropomyosin in extracts, IgE-binding protein profiles of the sensitized animals and IgE reactivity of tropomyosin were determined by immunoblot. Briefly, rTM, raw shrimp extract or boiled shrimp extracted were resolved on 10% SDS-PAGE and transferred to PVDF membrane with Trans-Blot Turbo (Bio-Rad). Non-specific binding was blocked with 5% non-fat dry milk, followed by incubation with serum pool comprising sera of eight mice per experimental group or sera of shrimp allergic subjects (Supplementary Table 1) at 1:10 dilution. Samples from mice with rTM-specific IgE > 0.34 OD, or OD of IgE_extract_ > OD of IgE_rTM_ on ELISA were selected. Membranes were then incubated with HRP-conjugated anti-mice IgE antibody, followed by SuperSignal West Pico PLUS Chemiluminescent Substrate (Thermo Fisher Scientific). Band signal was acquired with ChemiDoc MP Imaging system (Bio-Rad).

### Histological Analysis

Intestinal sample processing and histological staining were performed as per our previously published protocols [6, 15]. The entire intestine was collected, flushed by ice-cold PBS to remove luminal contents, filled with ice-cold 4% paraformaldehyde (PFA) and pre-fixed in PFA for 4 h. Intestine was then divided into three parts equally as duodenum, jejunum and ileum. Intestine segments were cut longitudinally and prepared according to the Swiss-roll method. The segments were fixed in 4% PFA overnight and embedded in paraffin. 4-µm tissue sections were stained with Naphthol AS-D chloroacetate esterase staining kit (Sigma Aldrich) to identify mucosal mast cells. Five randomly selected areas of the three intestinal segments were counted for each tissue sample. Mast cells were quantified per square mm using the software cellSens. Goblet cells were identified by periodic acid-Schiff (PAS) staining (Sigma Aldrich & Leica Microsystems) for detection of mucus-containing cells [16]. The number of goblet cells and epithelial cells were counted in ten randomly selected villi per sample. The percentage of goblet cells were expressed as the number of goblet cells divided by the total number of epithelial cells counted.

### Intestinal Cytokine Expression

Ileum sections were collected during tissue collection and stored in ice-cold RNAlater RNA Stabilization Reagent (Qiagen) immediately. Total RNA was extracted using TRIZOL (Invitrogen). The purity and concentration of purified total RNA were determined by measuring the absorbance at 260/280 nm ratio and 260 nm respectively. Total RNA was reversely transcribed using QuantiNova Reverse Transcription Kit (Qiagen) to synthesize cDNA. Quantitative real-time PCR was then performed to determine the expression of Th2-associated genes with specific primers, using the ABI7500 Fast Real-Time PCR system (Applied Biosystem) with QuantiNova SYBR Green PCR Kit (Qiagen). Relative quantification of mRNA expression was calculated by △△ cycle threshold method. The C_t_ (cycle threshold) value of each gene was normalized to C_t_ of house-keeping gene HRPT-1.

### Shrimp Allergic Subjects

Shrimp allergic subjects were recruited at the Prince of Wales Hospital, Hong Kong, with inclusion criteria of documented history of immediate allergic reactions within 2 h of shrimp consumption on at least two occasions over the past 5 years. Serum samples were collected at the time of recruitment during regular clinic visits for immunoblotting. Patients also underwent a double-blind placebo-controlled food challenge (DBPCFC) against black tiger prawn as described [17]. Skin prick test (SPT) was performed on day 1 before DBPCFC over the patients’ volar forearm with raw and heated shrimp extracts prepared in house (as described above), together with histamine (ALK-Abelló, 10 mg/ml) and normal saline as positive and negative controls respectively. Allergen-induced average wheal diameter (mm) was calculated as mean value of the longest and the midpoint orthogonal diameter of the wheal. SPT reaction was considered positive in case of a mean wheal diameter of 3 mm. Demographics of the subjects are shown in Supplementary Table 1. Participants in the study gave written-informed consent. Ethics approval was obtained from Joint Chinese University of Hong Kong—New Territories East Cluster Clinical Research Ethics Committee (no. 2018.484).

### Data Analysis

Intensity of IgE binding against tropomyosin on immunoblot was compared by densitometry analysis on Image Lab (Bio-Rad). The data were presented as mean ± SEM. Statistical significance of data was determined by one-way analysis of variance (ANOVA) followed by Kruskal–Wallis test, using Prism (GraphPad). Statistical difference of SPT wheal diameter was determined by paired t-test. The difference was considered as significant at a p value of < 0.05.

## Results

### Induction of Hypersensitivity Reactions

**Fig. 2:**
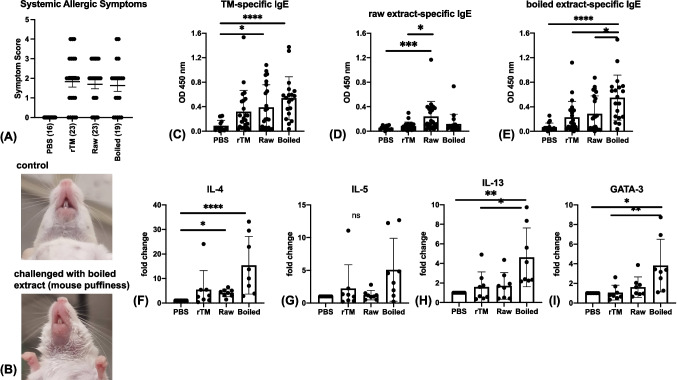
Allergic responses after intragastric challenge. (**A**) The responses of allergic symptoms: mice were evaluated 30 to 40 min after challenge and scored. The number of animals per group is showed in parentheses. (**B**) Images of representative reaction of negative control (PBS) and allergen (boiled extract) sensitized mice after challenge. Note the swelling of snout and mouth puffiness in the sensitized mouse but not in the PBS control mouse. Specific levels of IgE against (**C**) rTM, (**D**) raw shrimp extract and (**E**) boiled shrimp extract measured by ELISA. Relative mRNA expression of IL-4 (**F** IL-5), (**G**), IL-13 (**H**) and GATA-3 (**I**) of control and experimental groups (n = 8 per group). Data were normalized to HPRT-1. Data are expressed as mean ± SD. Data is considered statically significant when p < .05 on one-way ANOVA followed by Kruskal–Wallis test. * p < .05; ** p < .01; *** p < .001 and **** p < .0001

Systemic anaphylactic responses were observed for 30–45 min after the challenge on day 33 (Fig. 2A). Most sensitized mice exhibited allergic symptoms, varying from scratching from head to tail (score 1), puffiness around eyes and mouth (score 2, Fig. 2B) and increased respiration and reduced activities (score 3). Only mice in the tropomyosin and boiled shrimp groups showed the most severe allergic symptoms including tremors and no activities after prodding (score 4). More than 20% of sensitized mice from each group showed severe symptoms (score > 3) after challenge. No significant statistical difference was detected among the three experimental groups in the symptom scores.

### Induction of Th2 Cellular and Immunological Responses

Levels of TM-, raw extract- and boiled extract-specific IgE were measured by ELISA. Despite an increase in TM-specific IgE level in mice sensitized and challenged with rTM, only mice from the raw and boiled shrimp groups showed a significant increase in TM-specific IgE level comparing to the control mice (p = 0.01 and < 0.0001 respectively; Fig. 2C). Based on TM-specific IgE level and cut-off at 0.34 OD450nm, boiled shrimp extract had the highest success rate in sensitizing BALB/c mice (73.7%), followed by raw shrimp extract (47.8%) and rTM (34.8%). The mean level of TM-specific IgE was also slightly higher when induced by boiled extract (0.54 OD) comparing to raw extract (0.39 OD) and rTM (0.32 OD). Specific IgE levels to raw extract were comparatively low in all experimental groups, but raw extract induced significantly higher level of sIgE comparing to PBS (p = 0.0002) and rTM sensitization (p = 0.01, Fig. 2D). For specific IgE levels to the boiled extract, mice sensitized and challenged with boiled extract displayed significantly higher IgE level (mean = 0.55 OD) comparing to negative control (0.07 OD, p < 0.0001), rTM (0.23 OD, p = 0.022) and raw extract sensitized (0.29 OD, p = 0.045) groups (Fig. 2E).

Ileum sections were collected 24 h after challenge and extracted RNA and cDNA samples from eight mice with the highest IgE level from each group were selected for the measurement of Th2-associated gene expression (Fig. 2F-I). Expression of IL-4, IL-5, IL-13 and GATA-3 was most prominent in mice sensitized and challenged with boiled extract that was in line with the induction of specific IgE. The expression of IL-4 was 15-fold higher than the control group, while expression of IL-5, IL-13 and GATA-3 was 4-fold higher and statistically significant except for IL-5. Moreover, expression of IL-13 and GATA-3 was significantly higher in boiled extract group comparing to rTM group. However, mice in the rTM group did not show any remarkable up-regulation in these Th2 cytokines and transcription factor, while mice from the raw extract-sensitized group only showed significant up-regulation in IL-4 (p = 0.02).

### Changes in Intestinal Inflammatory Responses

**Fig. 3:**
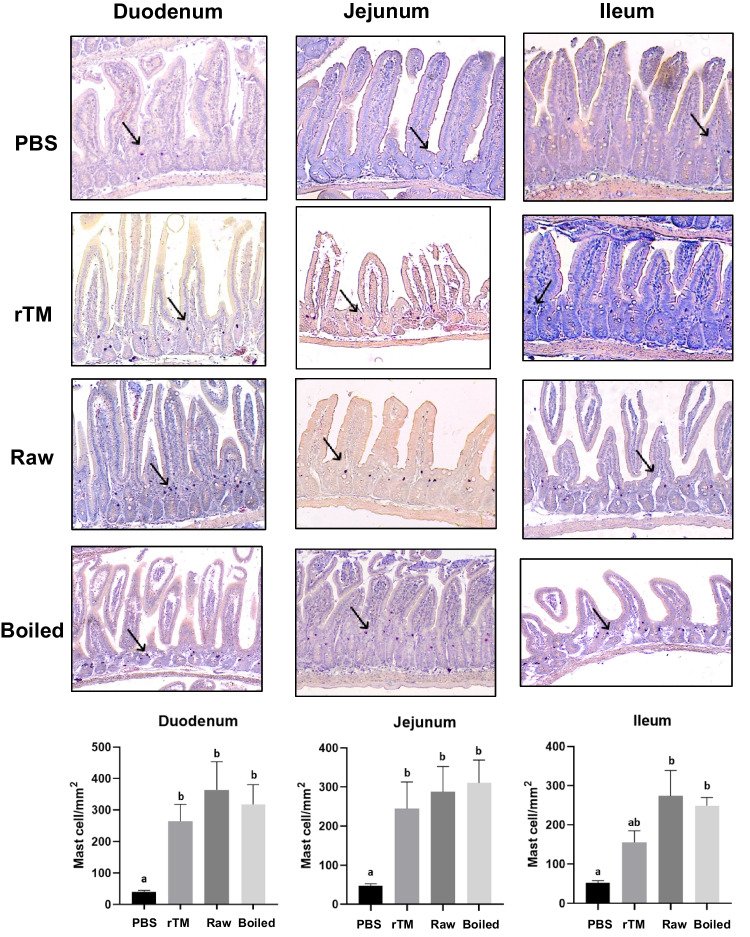
Mast cell infiltration in small intestine of sensitized and challenged animals. Representative sections of duodenum, jejunum and ileum (arrows indicate mast cells stained with chloroacetate esterase) of PBS control, rTM, raw and boiled extract groups. Quantification of mast cells in the three intestinal sections in control and experimental groups (n = 8) is shown in the bottom. Data are expressed as mean ± SD. Different letters indicate statistically significant differences. Data is considered statically significant when p < .05

**Fig. 4:**
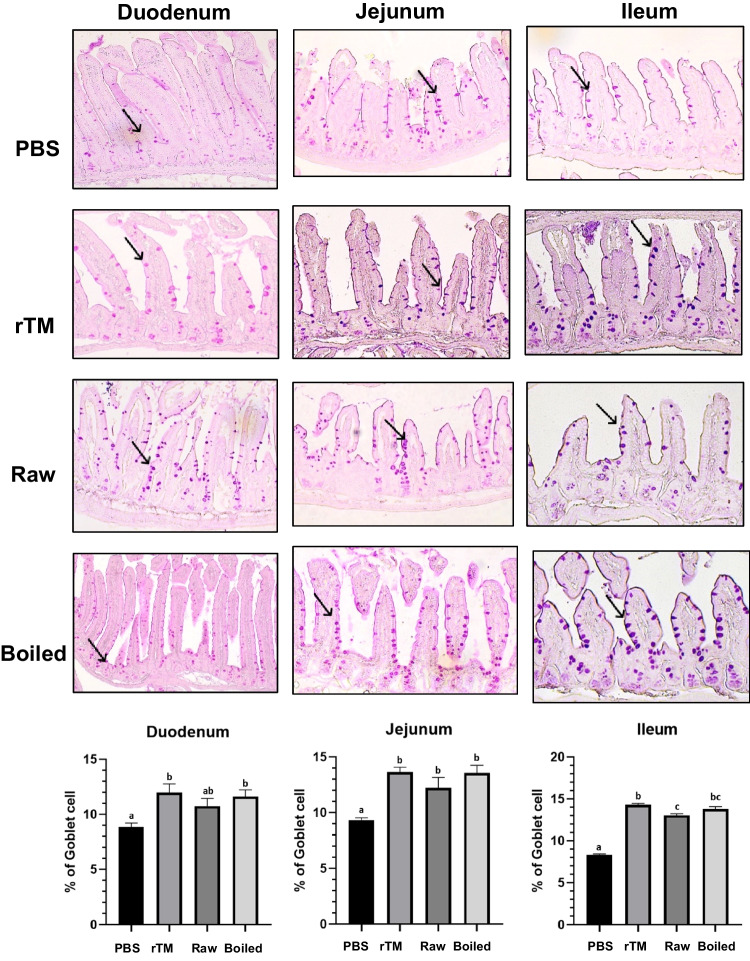
Goblet cell metaplasia in small intestine mucosa of sensitized and challenged animals. Representative sections of duodenum, jejunum and ileum (arrows indicate goblet cells stained in Periodic Acid-Schiff) of control and experimental groups. Percentage of goblet cells over epithelium cells in the three intestinal sections are also shown (n = 8). Data are expressed as mean ± SD. Data is considered statically significant when p < .05. Different alphabets indicate statistically significant differences

Intestinal samples were analyzed for inflammatory responses in eight mice with the highest IgE level from each group, including the number of accumulated mast cells at the mucosa crypt layer (Fig. 3) and the percentage of goblet cells in the villi of intestine (Fig. 4). In all three experimental groups, mast cell accumulation was most prominent in the duodenum, followed by jejunum and ileum. The number of mast cells increased significantly in all experimental groups when compared with the negative control group. There is no statistical difference between the three sensitization regimens in mast cell numbers and goblet cell hyperplasia.

### Comparison of Protein and Allergen Profiles

**Fig. 5:**
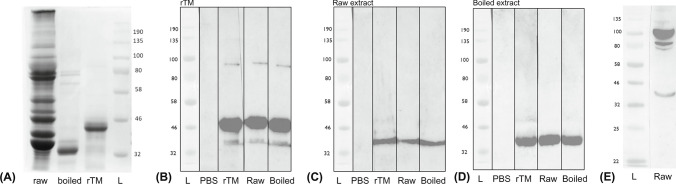
Protein and allergen profile. (**A**) 40 µg raw shrimp extract, 8 µg boiled shrimp extract, and 2 µg recombinant tropomyosin (rTM) were resolved on 10% SDS-PAGE. Note the significant loss of proteins in the boiled preparation. Immunoblot was performed with serum pool (n = 8) from PBS controls, rTM-, raw extract- and boiled-extract sensitized and challenge mice against (**B**) rTM (the 46 kDa protein band of his-tagged tropomyosin expressed in pET30a), (**C**) raw shrimp extract and (**D**) boiled shrimp extract. Note that IgE binding was only detected against tropomyosin in all experimental groups. (**E**) Pool of sera from raw extract-sensitized mice with higher IgE level to raw extract than to rTM (n = 5) were incubated against raw extract, and identified other shrimp allergens including hemocyanin (80 kDa) and glycogen phosphorylase (100 kDa). L, protein marker (New England Biolabs) with reference molecular weight (kDa) indicated

Our animal experiment data indicated a stronger sensitization capacity of boiled shrimp extract than raw extract and rTM. We therefore further analyzed the protein profile of the extracts and allergen profile with sera from sensitized animals. SDS-PAGE showed remarkable differences in protein profiles of raw and boiled shrimp extracts used in this study (Fig. 5A). Boiled shrimp extract showed a substantial loss in protein content, leaving only one major band at around 34 kDa. Immunoblot against rTM, raw and boiled extracts with sera from successfully sensitized mice (i.e. IgE_trop_ OD > 0.34, n = 8) was then performed. Tropomyosin was the only protein that exhibited IgE binding in all three experiment groups (Fig. 5B-D), suggesting that TM was the major allergen when shrimp extracts were used as the sensitizing agent. Meanwhile, we also noted that 4/23 mice sensitized by raw shrimp extract displayed positive raw extract-specific IgE but negative response to rTM or boiled extract, while one animal also had higher IgE to raw extract (1.17 OD) than to rTM (0.62 OD) and boiled extract (0.73 OD). These serum samples were also analyzed on immunoblotting against raw shrimp extract (Fig. 5E). Three distinct IgE-binding bands at 34 kDa, 80 kDa and 100 kDa were detected, which corresponded to the shrimp allergens tropomyosin, hemocyanin and glycogen phosphorylase, respectively.

### Allergenicity Comparison of Tropomyosin

**Fig. 6:**
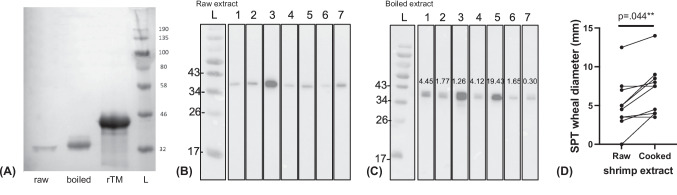
Comparison of tropomyosin allergenicity. (**A**) Immunoblot performed with sera from TM-sensitized mice against 40 µg raw shrimp extract, 8 µg boiled shrimp extract or 2 µg rTM. Immunoblot performed with sera from seven shellfish allergic subjects (1–7) against (**B**) raw shrimp extract and (**C)** boiled shrimp extract. Numbers in the lanes in (**C**) indicate the relative intensity of the IgE-binding bands in boiled extracted in comparison to the respective bands in raw extract (relative intensity = 1). Note the remarkably enhanced allergenicity of heat-treated TM. **(D)** SPT wheal diameter (mm) of ten DBPCFC-confirmed shrimp allergic patients. Statistical difference was determined by paired t-test (p = .044)

Noting that tropomyosin remains the major sensitizing allergen, we further compared the allergenicity of recombinant, untreated and heated TM. No statistical difference could be detected when comparing the specific IgE levels to rTM and boiled extract of rTM-sensitized mice, suggesting a comparable allergenicity of rTM and heated TM (Mann–Whitney test, p = 0.35). With sera of tropomyosin-sensitized mice, TM was detected in both extracts (~34–36 kDa) and purified rTM (~45–46 kDa) in immunoblot (Fig. 6A). It is of great importance that despite a 5-fold lower amount of protein being resolved on SDS-PAGE (Figs. 5A & 6A), IgE-binding capacity of TM was enhanced in the boiled extract with sera of sensitized mice comparing to raw extract. The relative allergenicity of untreated (raw) and heated (boiled) TM was further compared with sera of seven shellfish allergic patients by comparing the relative intensity of the immunoblot bands (Fig. 6B, C). Six out of seven samples showed enhanced IgE binding to heat-treated TM, and the band intensity (allergenicity) was 1.26- to 19.43-fold higher in heated TM comparing to raw TM (relative intensity defined as 1). In concordance with immunoassays, cooked shrimp extract induced larger SPT wheal diameter in ten DBPCFC-confirmed shrimp allergic patients than raw extract (paired t-test, p = 0.044) (Fig. 6D).

## Discussion

In this study, we established new mouse models of shrimp allergy with raw and boiled shrimp extracts based on our established animal model of tropomyosin-induced shrimp hypersensitivity. To our knowledge, this is the first in vivo study to illustrate the stronger allergenicity of the boiled shrimp extract with tropomyosin remaining as the major sensitizing allergen accounting for the manifestation of shrimp allergy. Our data also demonstrate the higher in vivo allergenicity of heat-treated tropomyosin comparing to raw tropomyosin, and reveals minor allergens relevant to clinical shrimp allergy.

Distinct from our previously reported recombinant tropomyosin-induced shrimp allergy model [5], the present experimental models use a combination of proteins extracted from shrimp muscle, either in raw or cooked forms, as sensitization and provocation agents. Our results show that shrimp extract presents higher immunogenicity than the purified allergen (rTM), denoted by the higher IgE sensitization rate and stronger Th2 inflammatory responses. This is consistent with similar studies using other food allergens, such as peach extract compared to using natural Pru p 3 [18], as well as using peanut extract compared to using Ara h 1, 2, 3 or 6 [19]. Purified Bet v 1 also lacks sensitizing potency without the bioactive proteins in birch pollen extracts [20]. It might be the result of the presence of Th2—promoting factors in the extracts and/or sensitization to multiple allergens triggering a more heterogenous pool of IgE antibodies. While our results illustrate tropomyosin as the major sensitizing allergen, the matrix effect of the shrimp extracts as an adjuvant to increase allergenicity, allergen availability and digestibility are factors that may account for the more prominent inflammatory responses in mice sensitized by shrimp extracts [21]. Other studies have also illustrated that the inner cavities of lipid transfer proteins, such as Pru p 3 and Tri a 14 (wheat allergen), allow them to accommodate different ligands that act as adjuvant in the allergic sensitization process [22]. The phytosphingosine (PHS) of these ligands also mimics the role of human inflammatory mediator sphingosine-1-phosphate that accounts for the association between LTPs sensitization and more severe allergic reactions. Ruiter et al. [12] also reported that peanut proteins induced unique gene expression in human myeloid dendritic cells including the gene encoding retinaldehyde dehydrogenase 2 (RALDH2) and aldehyde dehydrogenase 1 family, member A2 (ALDH1A2) that was not induced by purified peanut allergens single or combined, which in turn promoted the downstream production of retinoic acid and IL-5. These studies point to the potency of peanut proteins in inducing severe and life-long allergic responses. Our future study will explore the molecular factors and their Th2-inducing mechanisms to further understand the potent allergenicity of shellfish.

Unlike other “big 8” food allergen sources such as peanut and tree nuts, crustacean shellfish are reported allergic in raw, cooked and processed forms, and can even trigger anaphylaxis through the inhalation route [23]. It is therefore logical that different preparations of shellfish/shrimp could lead to varying sensitization profiles and thus the phenotype and manifestation of allergic reactions. In this study, immunoblot results indicate that TM remains the major sensitizing allergen in our extract-based animal models. This is in concordance with the sensitization profile of 80% of allergic subjects, and that tropomyosin is a good predictor of shrimp allergy [24]. Our data on cooked shrimp extract also illustrated that shrimp tropomyosin is a heat-stable allergen. But perhaps more importantly, we illustrated that hemocyanin and glycogen phosphorylase are important shrimp allergens beyond tropomyosin, although only a minority of raw extract-provoked animals (5/23, 21.7%) produced IgE against these allergens. These animal findings are congruent with patient data, as we reported previously that sensitization to glycogen phosphorylase (Pen m 14) was predictive of shrimp allergy [11]. Hemocyanin was also shown to be a major molecular cause of anaphylaxis due to shrimp cephalothorax and sensitization to hemocyanin is strongly associated to shrimp allergy in Spain, Italy and the United States alongside with tropomyosin [25, 26].

We are aware that cholera toxin was used to stimulate Th2 responses to overcome the tendency of developing oral tolerance against ingested antigens such that our models may just artificially mimic human disease and not fully reflect the pathology of allergic reactions in human. A recent study also demonstrated that Ara h 2 sensitization led to strong and sustained production of IgE only in C3H mice but not BALB/c that highlights strain-dependent differences [27]. Nevertheless, our models closely mimic the natural course of shrimp allergy in human presented with hallmark features of Th2 immunological changes and IgE binding profile. The models are thus valuable tools to provide substantial knowledge on the capacity of different shrimp allergens and proteins to cause sensitization.

Indeed, in our study, the boiled extract was a better inducer of shrimp allergy in BALB/c mouse model compared to the raw preparation, based on a better success rate in sensitizing the animals, higher allergen-specific IgE titer and more prominent Th2-skewed cellular responses. This is in line with our clinical results that boiled shrimp extract triggered larger wheal size in DBPCFC-confirmed shrimp allergic patients. The results are coherent with the report by Carnés et al. that boiled shrimp extract identified more patients and wheal sizes of the skin text were significantly higher than raw extract in 78 patients with reported allergic reactions upon seafood ingestion [28]. As illustrated by immunoblot assay, reasons for this can be partly attributed to the higher IgE-binding capacity of heat-treated tropomyosin than its native (raw) counterpart. TM has been well-described to withstand heat and high-pressure processing and retain its IgG/IgE reactivity. Based on structural analysis, the significant reduction in α-helix and β-sheet contents in heat-treated TM contribute to the remarkable increase in its IgE binding capacity comparing to raw TM in independent studies [29, 30]. Such structural alterations may lead to the unmasking of IgE epitopes giving rise to more accessible surfaces and/or increased IgE binding due to changes in the confirmational epitopes [10]. Herein we further provide robust in vivo and in vitro evidence to the higher allergenicity of heat-treated TM from both animal experiments and shrimp allergic patients. Such data might provide a further explanation to the potency of shellfish in triggering anaphylaxis and highlight the importance of refining our diagnostic strategy to include boiled extract, and perhaps heat-treated TM, to diagnose shellfish allergy.

In summary, we have demonstrated that intragastric administration of shrimp extracts, particularly heated, more effectively sensitizes BALB/c mice in terms of reactivity to recombinant tropomyosin. Thus, these two new murine models of shrimp extract-induced shrimp allergy provide valuable tools for better evaluation of novel therapeutic interventions and further our understanding of the mechanisms in shellfish allergy. We also provided robust in vivo clinical and animal data with respect to the stronger immunogenicity of shrimp extracts and heat-treated tropomyosin. It is therefore important to consider the use of heated extract/allergen in animal models when studying food allergen sources that can trigger reactions in both raw and cooked forms. These findings also underscore the inclusion of other shrimp allergens (hemocyanin and glycogen phosphorylase), as well as heated shellfish extract/tropomyosin in the diagnosis of shellfish allergy.

### Supplementary Information

Below is the link to the electronic supplementary material.

Supplementary file1 (DOCX 66 KB)

## Data Availability

No datasets were generated or analysed during the current study.
