# Supplementary material for: Shrimp Extract Exacerbates Allergic Immune Responses in Mice: Implications on Clinical Diagnosis of Shellfish Allergy
Source: Clin Rev Allergy Immunol. 2024 May 22;66(2):250–9. doi: 10.1007/s12016-024-08994-4 (PMC11193834; doi:10.1007/s12016-024-08994-4)
Supplement: Supplementary file 1 [file 12016_2024_8994_MOESM1_ESM.docx]

Supplementary Table 1 Demographics of shrimp allergic subjects

				SPT (mm)		ImmunoCAP sIgE (kUA/L)					BAT (%CD63+)	
Subject	Sex	Age (y)	DBPCFC	shellfish	HDM	f24 shrimp	f351 rPen a 1	f23 crab	f207 clam	f338 scallop	shrimp extract	Clinical History
1	M	31	A	4	5.5	6.52	3.29	5.88	0.9	1.74	88.7	I,A,U,V
2	M	2	ND	0	4	29.1	4	4.5	0.55	17.7	ND	A,R,V
3	F	5	ND	2.5	2.5	25.5	23.6	23.1	6.21	9.37	ND	A,V
4	F	4	ND	5	6	17.4	14.6	11.5	1.6	2.43	ND	U
5	F	46	ND	8.5	8.5	5.46	4.89	3.8	0.61	1.61	ND	I,A,U,SOB
6	M	11	ND	ND	ND	6.76	4.05	3.9	2.61	2.47	ND	U
7	M	28	ND	4.5	3	4.68	3.04	3.86	0.55	0.58	ND	I,A,U,SOB
8	F	23	A	0	0	0.11	0.04	0.06	0.05	0.11	1.9	A,U,R
9	M	12	A	9	12.5	1.96	2.12	1.21	0.39	0.66	80.3	I,A,U,R
10	M	22	A	2	5	12.1	0.04	4.92	0.23	0.44	78	A,U
11	F	19	A	0	2	1.2	0	0.83	0.02	0.01	18.4	U,R
12	M	30	A	0	5	0.1	0.07	0.53	0.05	0.12	49.5	A,U,SOB
13	F	30	A	5	ND	0.24	0	0.09	0.01	0.04	ND	I,U,W
14	F	19	A	4	8	8.46	7.04	7.04	3.99	4.85	ND	I,A,SOB
15	M	45	A	6.5	10.5	1.79	0.43	1.93	0.43	0.04	41.5	I,A,U
16	M	31	A	0	3.5	2.85	0.03	14.6	0.06	0.08	21.2	I,A,U,R,V
17	M	31	A	4	5.5	6.52	3.29	5.88	0.9	1.74	88.7	I,A,U,V
18	F	48	A	7.5	8.5	2.99	2.69	2.66	0.37	0.38	32.4	I,A,SOB
19	F	47	A	0	2	0.03	0.01	0.02	0.03	0.04	11.5	A,U
20	F	42	A	3.5	4	6.6	5.56	4.57	0.58	1.79	81.9	I,A,R,SOB
21	M	25	A	3	5	4.16	0.13	3.56	0.33	38.1	32.1	I,A,U,R,SOB
22	M	34	A	3	6	15.1	0.04	12.3	0.08	0.08	64.7	A,U,R,V

SPT, skin prick test; sIgE, specific IgE; BAT, basophil activation test; DBPCFC, double-blind placebo-controlled food challenge; y, years; HDM, house dust mite; M, male; F, female; ND, not done; I, itchy month/skin; A, angioedema; U, urticaria, V, vomiting; SOB, shortness of breath; W, wheeze.
